# Bidirectional Selection for Body Weight on Standing Genetic Variation in a Chicken Model

**DOI:** 10.1534/g3.119.400038

**Published:** 2019-02-08

**Authors:** Mette Lillie, Christa F. Honaker, Paul B. Siegel, Örjan Carlborg

**Affiliations:** * Department of Medical Biochemistry and Microbiology, Uppsala University, Uppsala, Sweden; † Department of Animal and Poultry Sciences, Virginia Polytechnic Institute and State University, Blacksburg, Virginia

**Keywords:** Chicken, White Plymouth Rock, selective sweeps, body weight, quantitative trait

## Abstract

Experimental populations of model organisms provide valuable opportunities to unravel the genomic impact of selection in a controlled system. The Virginia body weight chicken lines represent a unique resource to investigate signatures of selection in a system where long-term, single-trait, bidirectional selection has been carried out for more than 60 generations. At 55 generations of divergent selection, earlier analyses of pooled genome resequencing data from these lines revealed that 14.2% of the genome showed extreme differentiation between the selected lines, contained within 395 genomic regions. Here, we report more detailed analyses of these data exploring the regions displaying within- and between-line genomic signatures of the bidirectional selection applied in these lines. Despite the strict selection regime for opposite extremes in body weight, this did not result in opposite genomic signatures between the lines. The lines often displayed a duality of the sweep signatures, where an extended region of homozygosity in one line, in contrast to mosaic pattern of heterozygosity in the other line. These haplotype mosaics consisted of short, distinct haploblocks of variable between-line divergence, likely the results of a complex demographic history involving bottlenecks, introgressions and moderate inbreeding. We demonstrate this using the example of complex haplotype mosaicism in the *growth1* QTL. These mosaics represent the standing genetic variation available at the onset of selection in the founder population. Selection on standing genetic variation can thus result in different signatures depending on the intensity and direction of selection.

Initial thinking on how adaptive processes shape the genome was modeled by Smith and Haigh (1974), who demonstrated that a beneficial mutation favored by natural selection will increase in frequency within a population. Linkage disequilibrium in the flanking region of a selected allele will result in a characteristic valley of diversity around the selected variant, known as a hard selective sweep (Kaplan *et al.* 1989; Stephan *et al.* 1992). Aside from hard selective sweeps, selection on recessive variants, variants contributing to the standing genetic variation in a population, and partial sweeps (Hermisson and Pennings 2005; Teshima and Przeworski 2006) typically have weaker effects. Empirical studies, however, suggest that this type of selection is the most abundant mode of adaptation in recent evolution in both *Drosophila melanogaster* (Garud *et al.* 2015) and humans (Pritchard *et al.* 2010; Hernandez *et al.* 2011; Schrider and Kern 2017). Furthermore, polygenic adaptation describes how selection acts on standing genetic variation across the many loci contributing to a quantitative polygenic trait leading to a new phenotypic optimum by way of modest allele frequency changes across these loci (Pritchard and Di Rienzo 2010; Pritchard *et al.* 2010). Although this mode of adaptation would respond very rapidly to changes in the selective environment, it would not necessarily lead to fixation for any one variant (Pritchard *et al.* 2010).

Modes of adaptation are not mutually exclusive, and the genomic signature that results will be dependent upon factors such as the genetic architecture of the trait, standing genetic variation available within this architecture, effective sizes of standing haplotypes, and population demography. By exploiting population genetic signals, researchers are increasingly able to detect the underlying modes of selection, from initial sweep scans that identify valleys of low diversity resulting from hard sweeps, to various recent developments to detect and differentiate between soft and hard sweeps (Berg and Coop 2014; Garud *et al.* 2015; Schrider and Kern 2017). Whereas previous studies have attempted to uncover selection throughout human history (Coop *et al.* 2009; Schrider and Kern 2017), much can be learned from research with model organisms, such as selection in experimental populations of *Drosophila* (Burke *et al.* 2010) or mice (Chan *et al.* 2012). In particular, long-term selection experiments have well-defined population histories, likely have stronger selection signatures in the genome due to an isolation of the trait under selection, and allow breeding of crosses to test for adaptive trait associations to candidate sweeps. The Virginia body weight chicken lines, whose history of long-term, single-trait, bi-directional selection from a common founder White Plymouth Rock population has been well-characterized, affords us the opportunity to dissect the genomic selective-sweep signatures of strongly selected loci (Figure 1).

**Figure 1:**
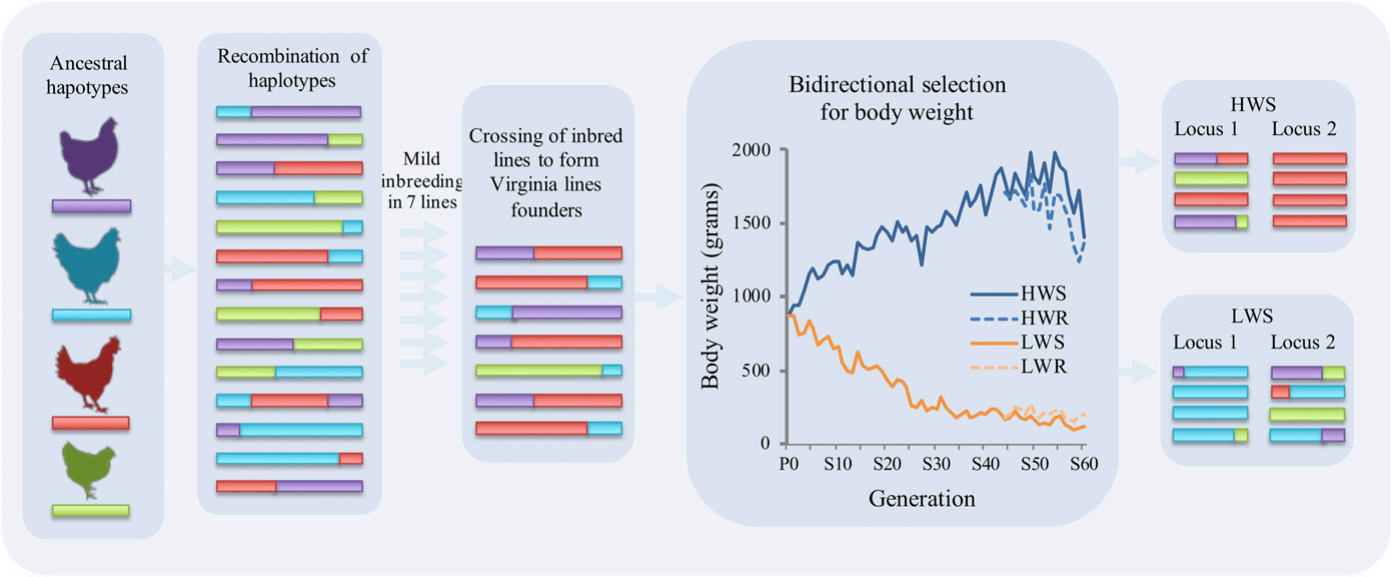
Representation of the formation of genomic signatures in the Virginia body weight lines. Ancestral haplotypes that contributed to the formation of the White Plymouth Rock breed would have recombined over time. Haplotype variation was likely constrained in the seven partially inbred lines, which were crossed to form the founder population of the Virginia body weight lines. The bidirectional change in eight-week body weight in the Virginia body weight lines is presented in the graph from the parental (P0) across the selected generations (S1-S60) for the high weight selected (HWS; blue unbroken line) and low weight selected (LWS; orange unbroken line) body weight lines. Body weight within relaxed sublines are also shown for the high weight relaxed line (HWR; blue broken line) and low weight relaxed line (LWR; orange broken line) at corresponding selected generation. Haplotype frequency and ancestral haplotype origin are depicted at two hypothetical loci to the right. At locus 1, HWS continues to segregate with multiple haplotypes, whereas LWS is fixed for an extended blue haplotype. At locus 2, HWS is fixed for an extended red haplotype, whereas LWS continues to segregate with multiple haplotypes.

Founders for the Virginia lines were generated via crossing of seven partially inbred White Plymouth Rock lines, in effect constraining the standing diversity. From this base population in 1957, bidirectional selection for 8-week body weight was carried out to form the high (HWS) and low (LWS) Virginia body weight lines. The breeding regime structured to minimize inbreeding and minimize the stochastic fixation of alleles that could affect small breeding populations (Siegel 1962; Marquez *et al.* 2010). Relaxed sublines for both HWS and LWS were produced from selected generation 44 (high weight relaxed: HWR; low weight relaxed: LWR) (Dunnington *et al.* 2013). After 55 generations of divergent selection, a 15-fold difference in body weight exists between the lines (Jambui *et al.* 2017b). With its well-defined population history, bi-directional single trait selection regime, and well-defined polygenic architecture of the adaptive trait, the Virginia body weight lines represent an invaluable resource to investigate the genomic signatures of selection.

Previous research has demonstrated that standing genetic variations in many loci from the founder population of the Virginia lines contribute to the observable difference in body weight. Sweep scans using individual genotypes from a 60k SNP-chip have revealed numerous regions of differentiation between the selected lines (Johansson *et al.* 2010; Pettersson *et al.* 2013). These were explored for associations with body weight and a large number of them were found to have contributed to selection response using available standing genetic variation (Sheng *et al.* 2015). Using genotype data from these sweeps and earlier identified QTL, together with phenotypic data from an F_15_ intercross between the lines, 20 independent associations to 8-week body weight have been confirmed (Zan *et al.* 2017).

Recently, pooled genome resequencing was applied to several generations from these lines to investigate whether these data could reveal any additional features of the genomic impacts of bidirectional selection on body weight to those previously found using the 60k SNP-chip data. An initial evaluation of these data were integrated into a review of the knowledge gained from multiple lines of inquiry in the Virginia body weight lines, with a particular focus on identified QTL regions (Lillie *et al.* 2018). Here, performed is a more in-depth analysis of this pooled genome resequencing dataset to characterize the genomic signatures of highly differentiated regions across the whole genome between the bidirectionally-selected lines: the putative selective sweeps. As earlier shown for the QTL regions (Lillie *et al.* 2018), between-line differentiation was seldom due to complete fixation of different haplotypes in the lines. Instead, most selective sweeps resulted from extended runs of homozygosity in one line, contrasting to persistence of heterozygosity in the other. This duality of selection signatures and haplotype structures was illustrated by dissecting the complex differentiation in an earlier identified QTL on chromosome 1. Typically, this heterozygous region was comprised of multiple, distinct regions, with variable diversity, and between-line divergence. The directions of these relationships do not suggest any line bias, suggesting that the physiological plateau since generation 35 in the LWS due to a disruption of food-consumption and an inability to enter egg production at less than 1000 g (Siegel and Dunnington 1985; Siegel and Dunnington 1987; Jambui *et al.* 2017a) has not had a major influence on these patterns.

These empirical observations and our knowledge about the history of these populations imply that the duality of these signatures genome-wide reflect positive selection for one large-effect haplotype in one line while in the other line negative selection would remove this haplotype, allowing other haplotypes to continue to segregate. Mosaic haplotype structures within these segregating regions reflect standing variation in the founder population, likely resulting from ancestral haplotype recombination along a history of bottlenecking, inbreeding, and crossbreeding.

## Materials and Methods

### Virginia body weight chicken lines

All animal procedures were carried out by experienced handlers and in accordance with the Virginia Tech Animal Care Committee animal use protocols (IACUC-15-136). The Virginia body weight lines were formed from a founder population resulting from crossing seven lines originating in 1949 that had undergone mild inbreeding. Established in 1957, bidirectional selection for body weight at 8 weeks of age was initiated to produce the closed selected lines: high weight selected (HWS) and low weight selected (LWS) (Siegel 1962). Breeding focused on a response to selection, while attempting to minimize inbreeding (Marquez *et al.* 2010). Effective population sizes in the LWS and HWS lines have been estimated as 38.3 and 32.1, respectively (Marquez *et al.* 2010). Relaxed sublines for both HWS and LWS were produced from selected generation 44, and are referred to as high weight relaxed (HWR) and low weight relaxed (LWR) (Dunnington *et al.* 2013). All generations were hatched in the same incubators and reared in the same pens on the same diet. Pooled semen was used to produce each generation of relaxed lines.

### Sequencing and genome alignments

The sequencing data used in this dataset was originally reported in (Lillie *et al.* 2018). In short, DNA for the genomic analyses was prepared from blood samples collected from 9-30 individuals from each line and pooled in equimolar ratios prior to library construction. Genome sequencing library construction and sequencing was carried out by SciLifeLab (Uppsala, Sweden) using two lanes on an Illumina Hiseq 2500. Reads were aligned to the *Gallus gallus* genome (Galgal5; INSDC Assembly GCA_000002315.3, Dec 2015) using BWA (Li and Durbin 2009). Genomes were sorted and duplicates were marked and removed with Picard (v1.92; http://picard.sourceforge.net). GATK (v3.3.0; McKenna *et al.* 2010) was used for realignment around indels. GATK UnifiedGenotyper was used to generate allele calls at all sites (option: emit all sites) and with ploidy = 30 (18 for LWS generation 50 as only 9 individuals went into this pool) to account for the pooled genome sample. Sites were filtered to only include those with >10 and <100 reads, wherefrom allele frequency, heterozygosity, and pairwise *F_ST_* between all populations were calculated. Samtools (Li *et al.* 2009; v1.1; Li 2011) was used to generate mpileup files for PoPoolation2 (v1.201; Kofler *et al.* 2011), which was used to calculate *F_ST_* over 1000 bp sliding windows with 50% overlaps between the population samples using the Karlsson *et al.* (2007) method, with minimum count 3, minimum coverage 10, maximum coverage 100, and minimum coverage fraction 1. Genome alignments were visualized in IGV (v2.3.52; Robinson *et al.* 2011; Thorvaldsdottir *et al.* 2013).

### Differentiated regions

As reported in (Lillie *et al.* 2018), differentiated regions were identified by employing an empirical *F_ST_* threshold of 0.953, representing the top 5% *F_ST_* values in generation 55. Windows with *F_ST_* values above this threshold were clustered into differentiated regions when they were less than 100 kb from one another. Clusters with less than 2 SNPs or less than 100 kb were removed from the dataset to retain only the stronger candidate regions. Mean and median heterozygosity were calculated for each line within each differentiated region. We used the Variant Effect Predictor (VEP) (McLaren *et al.* 2016) available from Ensembl (Aken *et al.* 2017) to investigate potential functionality of candidate alleles. Haplotype structure within regions of interest were visualized using adjusted allele frequencies (Lillie *et al.* 2017) (similar to the allele polarization step in the haplotype-block reconstruction approach used by Franssen *et al.* 2017). This approach adjusts allele frequencies within the sequenced lines to the generation of lowest haplotypic complexity, such that allele frequencies across all lines would be adjusted to 1-AF, for sites where allele frequency > 0.5 in the generation of lowest haplotypic complexity. In most cases, the generation of lowest haplotypic complexity contains one fixed extended haplotype within the region of interest, with raw allele frequencies equal to ∼0 or ∼1, generating adjusted allele frequencies equal to ∼0. These were then plotted using custom R scripts.

### Data availability

Pooled genome data generated for this study are available via Sequence Read Archive (SRA, https://www.ncbi.nlm.nih.gov/sra) under bioProject: PRJNA516366; bioSample: SAMN10787895; and accessions: SRR8480632-SRR8480641. Supplemental material available at Figshare: https://doi.org/10.25387/g3.7674281.

## Results

**Figure 2:**
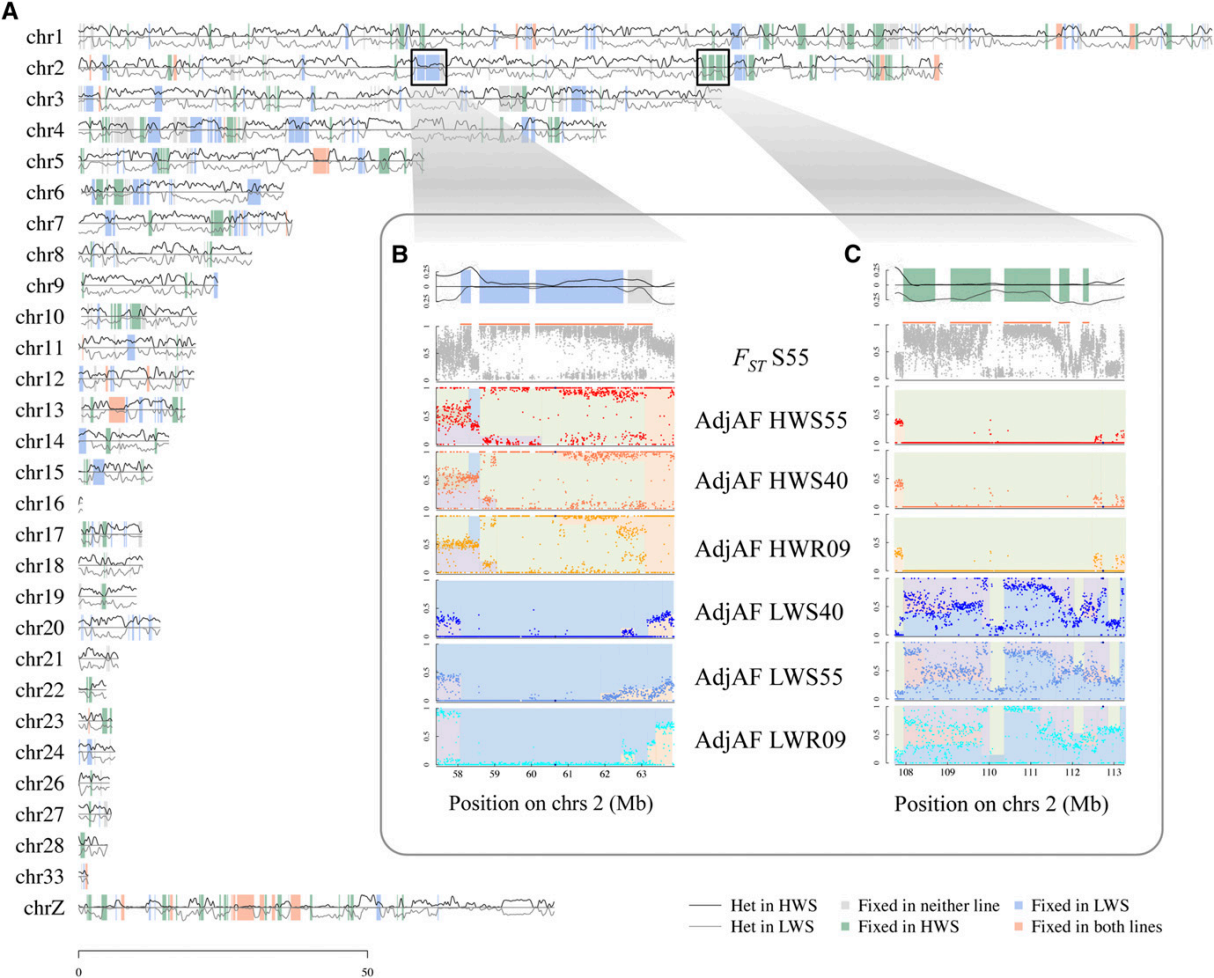
Heterozygosity across chromosomes of divergently selected Virginia body weight chicken lines. A. Heterozygosity in generation 55 for high weight selected (HWS; black line above x-axis) and low weight selected (LWS; gray line below x-axis) presented across the chicken chromosomes within the differentiated regions shaded using color code: gray where neither line is fixed (); green where only HWS is fixed (); blue where only LWS is fixed within the differentiated region; red where both lines are fixed. B. *F_ST_* and heterozygosity patterns across chromosome 2:58-63 Mb for selected generations 55 and 40 and relaxed generation 9. C. *F_ST_* and heterozygosity patterns across chromosome 2:108-113 Mb for selected generations 55 and 40 and relaxed generation 9. Panels within B and C insets: Panel 1: Detail of heterozygosity trace from chromosome map. Panel 2: Mean *F_ST_* within 1 kb windows between HWS and LWS at generation 55 indicated with gray points; region of differentiation indicated with the orange line above *F_ST_* plot; allele frequencies of SNP markers with association to body weight indicated with blue diamonds. Panel 3-8: Mean adjusted allele frequency (adjAF) of 5kb windows in HWS generation 55 (HWS55) / HWS generation 40 (HWS40) / high weight relaxed generation 9 (HWR9) / LWS generation 55 (HLWS55) / LWS generation 40 (LWS40) / low weight relaxed generation 9 (LWR9). Shaded colors within these plots have been used to highlight the runs of adjusted allele frequencies that contribute to different haploblocks.

Alignment coverage of the reference genome after read alignment was between 91.22% and 91.44% across the sequenced pools. As reported earlier (Lillie *et al.* 2018), 395 differentiated regions between the HWS and LWS lines in selected generation 55 were identified from the clusters of high differentiation (*F_ST_* > 0.953; Figure 2). These regions covered a total of 174.5 Mb, or 14.2% of the genome, which is an increase from the 244 differentiated regions identified in selected generation 40 (99.6 Mb or 8.1% of the genome; Figure S1).

### Candidate selective sweeps are often polymorphic in one of the selected lines

**Figure 3:**
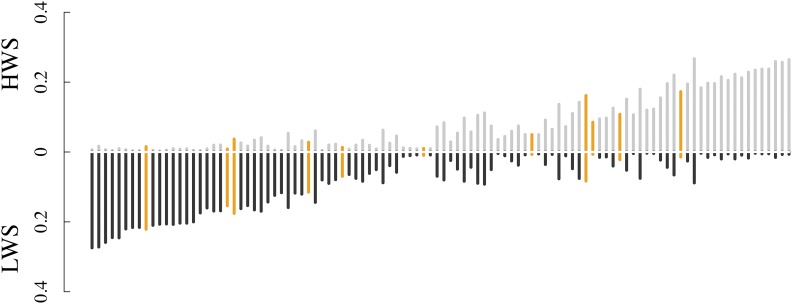
Mean heterozygosity in HWS (above the x-axis; gray) and LWS (below the x-axis; black) at generation 55 within differentiated regions greater than 0.5 Mb in length. Differentiated regions overlapping known associations with 8-week body weight (Zan *et al.* 2017) are indicated in orange. Regions presented are sorted in order of increasing cumulative heterozygosity across both lines with increased heterozygosity in HWS.

Compared to the genome-wide trend, there was a decline in heterozygosity at the extremes of high FST (S2 Figure). Few regions of differentiation showed fixation in both lines; rather, it was often the case that there was fixation across an extended region in one line, while many nucleotide positions in the region still segregated in the other (Figure 3; S3 Figure). Of the differentiated regions greater than 0.5 Mb in length, 34 regions (33%) were close to fixation in HWS (mean heterozygosity <= 0.1) while LWS continued to segregate (mean heterozygosity > 0.1), 33 regions (32%) were close to fixation in LWS (mean heterozygosity <= 0.1) while HWS continued to segregate (mean heterozygosity > 0.1), and 37 regions (35%) were close to fixation for alternative haplotypes in both lines (mean heterozygosity <= 0.1 in both). This demonstrated that, while one extended haplotype was fixed in one line, multiple haplotypes continue to segregate in the other. Fixation was as common in HWS as LWS, a trend that extended to regions with confirmed associations for the selected trait, 8-week body weight (Figure 3; S3 Figure) (Zan *et al.* 2017).

### Mosaic haplotypes in the growth1 QTL

**Figure 4:**
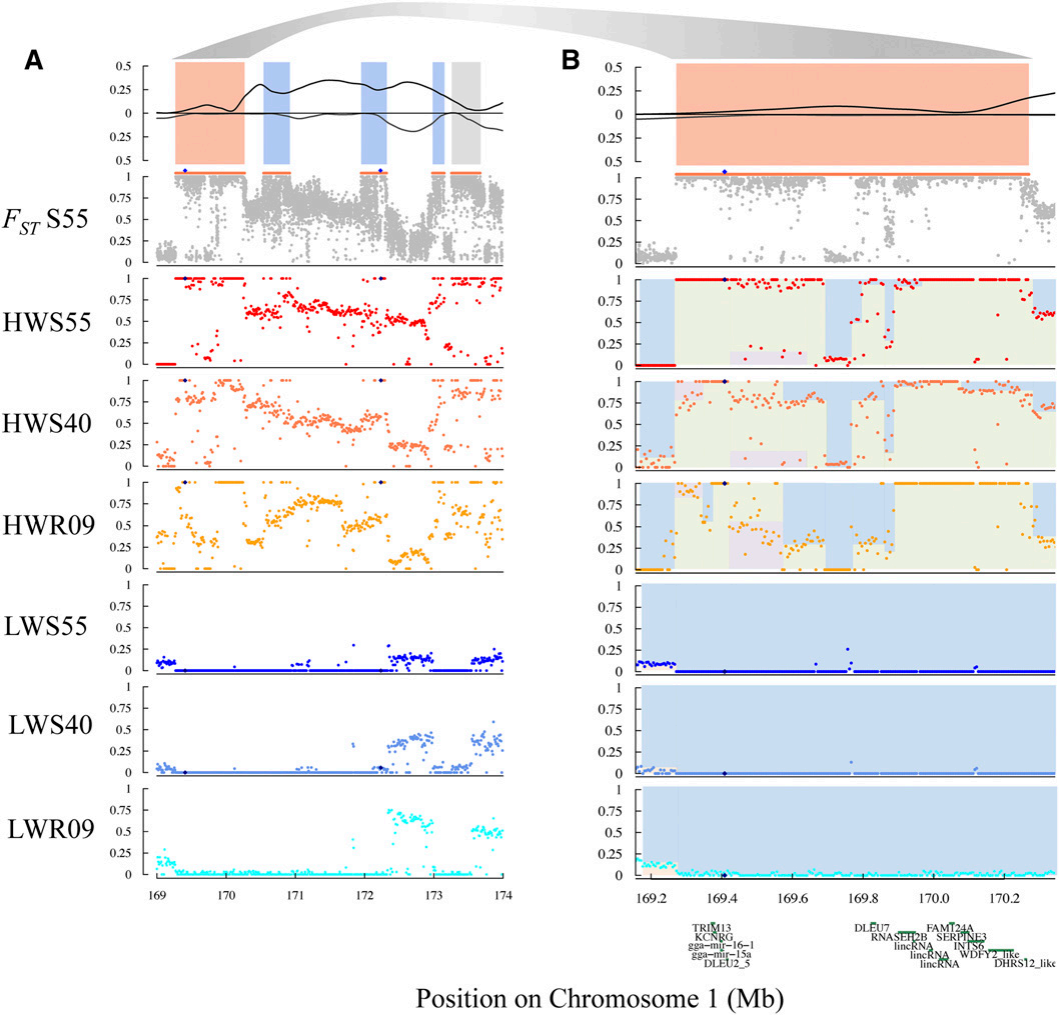
A. *F_ST_* and heterozygosity patterns across chromosome 1:169-174 Mb for selected generations 55 and 40 and relaxed generation 9 of the Virginia body weight chicken lines. B. *F_ST_* and heterozygosity patterns across chromosome 1:169.2-170.3 Mb for selected generations 55 and 40 and relaxed generation 9 with gene positions. Panel 1: Heterozygosity. Panel 2: Mean *F_ST_* within 1 kb windows (gray points) in selected generation 55; defined region of differentiation indicated with orange line above *F_ST_* plot; blue diamonds indicate allele frequencies of SNP markers with significant association to body weight from Zan *et al.* (2017) . Panel 3-8: Mean adjusted allele frequency of 5kb windows in HWS55 (panel 3), HWS40 (panel 4), HWR09 (panel 5), LWS55 (panel 6), LWS40 (panel 7), LWR09 (panel 8). Colors within the plots are used to highlight the runs of adjusted allele frequencies that contribute to different haploblocks.

To illustrate the genomic mosaicism observed in many differentiated regions, we investigated the *growth1* QTL in detail (Jacobsson *et al.* 2005; Zan *et al.* 2017). A long region of divergence was observed between 169.3 Mb and 173.7 Mb on chromosome 1 (in total 4.4 Mb), where a single extended haplotype was close to fixation in LWS by generation 40, whereas the pattern of polymorphism in the HWS suggest that multiple haplotypes segregate in this line (Figure 4). Approximately 28% of the nucleotide positions within this region were highly divergent between HWS and LWS at generation 55. In total, 10,148 from 36,934 sites had differences between the lines in allele frequency that were greater than 0.9. This level of divergence implies that the long haplotype fixed in LWS is not present in the HWS at generation 55 because it has been selected out.

We checked for the presence of a known insertion that has been previously associated with increased body weight in other chicken populations (Jia *et al.* 2016). By examining the soft-clipped reads (S4 Figure), we observed that this variant also segregated in our population within the short region fixed for divergent haplotypes in both LWS and HWS (approximately 50 kb in length; GGA1: 169,370,000-169,420,000). Contrary to the expectation that this insertion would dominate in the HWS line, it was instead present on the long haplotype fixed by generation 40 in LWS, and entirely absent in HWS. Furthermore, this insertion is linked to the LWS allele of SNP marker rs14916997, which was associated with low body weight (Zan *et al.* 2017).

To identify candidate linkages and thus other potential functional variants, the *growth1* QTL was evaluated for further sub-haplotype fixations. In the nearby region (GGA1: 169,950,000-170,220,000), the HWS and LWS were also fixed by generation 55. Bounded by these two highly differentiated regions are 15 annotated genes (S1 Table), with 8 missense mutations (S2 Table). One missense mutation with predicted (SIFT) deleterious effect was present in HWS, located within the sixth exon of Ribonuclease H2 Subunit B gene (*RNASEH2B*), as well as the small (18 bp) deletion in LWS at GGA1: 169,407,811, which was absent from HWS.

## Discussion

There was an overall increase (244 to 395) in the number of differentiated regions between the HWS and LWS from generation 40 to generation 55. This increase is consistent with the continuing phenotypic divergence between the lines resulting from the ongoing response to selection in the HWS (average 56-day body-weights 1264 g and 1506 g, respectively) contrasted to the plateau in the LWS at 142 g (Lillie *et al.* 2018).

### Candidate selective sweeps were often polymorphic in one of the selected lines

Within highly differentiated candidate selective sweep regions, we observed that an extended haplotype was often fixed in one line while multiple haplotypes continued to segregate in the other, thus resembling a duality in these sweep signatures. These patterns are likely to be shaped by the intensity of selection on the locus, as well as the haplotype frequencies that were present in the base population. For example, haplotypes with equal but opposite effects present at the same frequencies at the onset of selection would be expected to result in relatively equal lengths of fixation (homozygosity) in both lines, and thus a high *F_ST_*. This pattern was observed only a few times (Figure 3), and thus appears to have been a relatively rare event. More likely is that the haplotypes in the founder population had different effect sizes and were present at different frequencies when selection was initiated to develop the high and low body weight chicken lines. The long runs on homozygosity found only in one line within regions of differentiation therefore likely represent the haplotypes with the largest effect, with the signature possibly being amplified by being at a low frequency at the onset of selection. These patterns suggest that the effect size of functional variants, as well as founding haplotype frequencies, are likely to have influenced the selective signature in the genome of the Virginia body weight chicken lines. The lack of line-bias in these signatures suggest that the selection plateau reached in the LWS at about generation 35, that physiologically is due to a disrupted food-consumption in some birds and an inability to enter egg production at less than 1000 g (Siegel and Dunnington 1985; Siegel and Dunnington 1987; Jambui *et al.* 2017a), has not made any major impact on these patterns.

A challenge in selective sweep studies is to discriminate which differentiated regions are likely due to drift or selection. The influence of drift on these lines has been investigated in a previous study, which showed that the population size was sufficiently large to prevent genetic drift from overriding the effect of selection for the loci with the larger effects and that the probability of fixation for alternative haplotypes by drift was very low (Johansson *et al.* 2010). Many regions of differentiation are present both within and outside of those known to be associated with body-weight (Sheng *et al.* 2015; Lillie *et al.* 2018; Zan *et al.* 2017). Although it is known that the already associated regions only explain part of the variation in body-weight (Zan *et al.* 2017), the observation made here that fixation for alternative haplotypes is seldom the case even within regions of differentiation makes it difficult to quantify the expected influence of drift in shaping genomic signatures. By implementing a high *F_ST_* cutoff, integrating association mapping results from previous studies in these lines, and comparing gene ontology and associations from other chicken populations, we have endeavored to build more confidence in differentiated regions than can be obtained separately using the approaches.

### Mosaic haplotypes

Domestication of the chicken began roughly 7,000 years ago, predominately from red junglefowl, *Gallus gallus* (West and Zhou 1988; Sawai *et al.* 2010), but potentially also with contributions from *Gallus sonneratii* (gray) and *Gallus layafetii* (Sri Lankan) (Eriksson *et al.* 2008; Groeneveld *et al.* 2010; Tixier-Boichard *et al.* 2011). With subsequent distribution of chickens via human dispersal and trade, local breed formation would subject established chicken populations to genetic drift and selection to suit the environment and human-imposed selection for breed standards and production traits (Lyimo *et al.* 2014). Regardless of expectations that founder effects, bottlenecks, and selection would impact genetic diversity, nucleotide diversity and substitution rates were comparable for red jungle fowl and domestic chickens. This implies that domestication did not result overall in a substantial genome-wide loss of diversity in the species and had only a minor effect in an evolutionary context (Tixier-Boichard *et al.* 2011).

Thus, recombination of divergent haplotypes from European and Asian populations occurred while producing the WPR breed, forming the genetic substrate available for artificial selection for high and low body weight (Figure 1). During the several decades of WPR breeding that preceded the initiation of the Virginia body weight chicken lines, recombinant haplotypes are likely to have arisen, ultimately producing finely grained mosaics of the chromosomes entering the founders of the Virginia body weight lines. It is on this standing variation which the bi-directional selection experiment has acted (Figure 1). Short haplotype mosaics within the longer selective sweep regions were revealed when comparing the HWS and LWS genomes. The *growth1* QTL region on chromosome 1 is a clear example, illustrating the haplotype mosaicism of sweep signatures observed across the genome.

### Mosaic haplotypes in the growth1 QTL

The *growth1* QTL was first defined in a microsatellite-based analysis of an F_2_ cross between the HWS and LWS, with a length of almost 110 cM (Jacobsson *et al.* 2005). This QTL was confirmed in SNP-based analyses of the F_2_ (Wahlberg *et al.* 2009) and fine mapped in the F_2_-F_8_ (Besnier *et al.* 2011; Brandt *et al.* 2016) and the F_15_ generation of the Advanced Intercross Line (Zan *et al.* 2017). In this latest study, two associations were found within *growth1*, with the SNP rs14916997 (GGA1: 169,408,309) having the strongest association (Zan *et al.* 2017). From the pooled genome data, it is evident that a major haplotype, between 169.3 Mb and 173.7 Mb (in total 4.4 Mb), is close to fixation in LWS (Figure 4). This haplotype was already close to fixation by generation 40, possibly indicating that selection has been relatively strong, and the resulting pattern resembles a hard sweep in LWS. Contrastingly, multiple haplotypes segregate in HWS, with a mixture of nucleotide positions that are divergently fixed when compared to LWS, intermixed with positions that segregate for both LWS and unique HWS alleles.

We observed a high level of divergence between HWS and LWS within this region, implying that the long haplotype fixed in LWS is not present in the HWS at generation 55 because it was selected out of this line. Highly dissimilar regions are intermixed with short, but continuous, stretches where the HWS and LWS haplotypes are nearly identical. Additionally, the boundaries between these shared and differentiated regions are distinct. They likely represent historical recombination events shared between one or more selected haplotypes, rather than being multiple classic hard sweeps, as these are expected to result in a gradual breakdown of population-wide linkage disequilibrium.

This interpretation is supported by other observations. First, for sharing of such interspersed haplotypes to be possible between the lines, formative recombination events must have occurred prior to the onset of bidirectional selection. Second, as several shared haplotype segments were sometimes observed in the divergent regions, such multiple events must have happened on the same haplotypes. Third, because the shared regions are often short (10s to a few 100 kb), the events are unlikely in a population with as small a population-size as the Virginia body weight chicken lines (effective population size, N_e_ ∼35) (Marquez *et al.* 2010). Finally, if the recombination events occurred during the selection experiment, selection on the recombinant haplotypes must have been strong in order to only retain them in the lines. Such is unlikely given the highly polygenic genetic architecture of 8-week body weight and the dilution of selection pressure across the many loci. Therefore, these haplotype mosaics most likely represent recombinant founder haplotypes resulting from their population history, including the formation of the WPR as a breed. Although recombination cannot be completely ruled out as a contributor to haplotype mosaicism, without individual-based sequencing, we cannot confirm or eliminate a role for recombination events in HWS and LWS.

Positive selection of this long haplotype to fixation in the LWS, coupled with negative selection for its removal from HWS, may serve as an explanation for why previous studies have seen a transgressive effect on 8-week body weight for SNP markers within this region. Zan *et al.* (2017) reported that while the HWS allele at the rs14916997 SNP marker (GGA1:169,408,309 bp) was associated with an increased 8-week body weight (additive genetic effect approximately 26 grams), the HWS allele at the nearby SNP marker rs316102705 (GGA1: 172,235,208 bp) was associated with a decrease in 8-week body weight (additive genetic effect of approximately -7 grams).

Notably, this region on chicken chromosome GGA1 appears often in association studies carried out in other populations, including comb traits (Shen *et al.* 2016), egg weight (Yi *et al.* 2015), feed intake (Yuan *et al.* 2015), abdominal fat percentage (Abasht and Lamont 2007; Sheng *et al.* 2013), shank metrics (Sheng *et al.* 2013), and growth and body weight at numerous life stages (Xie *et al.* 2012; Sheng *et al.* 2013; Abdalhag *et al.* 2015; Zhang *et al.* 2015). This may reflect a shared ancestral variant that has spread in domestic populations due to its beneficial effect, or that this is a gene rich region associated with many functionally important genes. Recently, an insertion (GGA1: 169,399,420) located upstream of the *miR-15a-16* precursor was strongly associated with growth traits in an Xinghua & White Recessive Rock F_2_ cross, where presence of this variant results in an altered hairpin formation, reduction of *miR-16* expression, and increased body weight, bone size, and muscle mass (Jia *et al.* 2016). Although this insertion was also present in multiple chicken breeds, and at high frequencies in broiler breeds (Jia *et al.* 2016), in our population, the insertion was present in LWS on the long, fixed haplotype, and was absence from the HWS, contrary to expectations that the insertion would substantially increase body weight.

This observation may be explained by alternative hypotheses; *i.e.*, that this insertion: i) has an effect also in our lines but is linked to another polymorphism with a stronger opposite effect, ii) does not have an effect in our lines due to genetic background, iii) does not have an effect at all, suggesting that earlier reports revealed association between this polymorphism and the studied traits, rather than causation. Alternatively, as is often the case, the functional variant may lie outside coding regions. Nevertheless, further work within this QTL region will be required to fully characterized the functional variant responsible for the large difference in 8-week body weight in the Virginia body weight lines.

## Conclusions

Over the course of long-term bidirectional selection, the Virginia body weight lines have experienced significant changes impacting behavioral, neurological, metabolic, and developmental processes (Dunnington and Siegel 1996). With a concerted effort to understand the fundamental genomics underlying these changes, we have employed linkage and association mapping, selective-sweep analyses using high-density SNP data and pooled genome sequencing across generations of the selected lines, relaxed lines, and a derived advanced intercross line (Wahlberg *et al.* 2009; Johansson *et al.* 2010; Pettersson and Carlborg 2010; Besnier *et al.* 2011; Pettersson *et al.* 2011; Ahsan *et al.* 2013; Pettersson *et al.* 2013; Sheng *et al.* 2015; Brandt *et al.* 2016). It has become clear that the difference in body weight between the selected lines relies on small to moderate effects from many loci, with the most recent analysis revealing 20 contributing loci (Zan *et al.* 2017).

Pooled genome resequencing revealed distinct hallmarks of selection in regions that were highly differentiated in the lines after 55 generations of long-term experimental selection. More often than not, the regions show the persistence of haplotypic diversity in one line, contrasted by fixation in the other, as demonstrated in the *growth1* QTL region. Despite selection acting on the same pool of standing genetic variants, the genomic signatures of selection thus resemble classic hard sweeps in one line, contrasted to a mosaic pattern of divergence in the other. This haplotype mosaic emerges from recombination of multiple divergent founder haplotypes, probably shaped by historical bottlenecks, crossbreeding, and inbreeding, which forms the standing genetic variation available in this selection experiment.
